# Primary cilia and cancer: a tale of many faces

**DOI:** 10.1038/s41388-025-03416-x

**Published:** 2025-04-29

**Authors:** Rebecca Collinson, Barbara Tanos

**Affiliations:** Centre for Genome Engineering and Maintenance, Department of Biosciences, College of Health, Medicine and Life Sciences, Brunel University London, Uxbridge, London UK

**Keywords:** Cancer, Biomarkers

## Abstract

Cilia are microtubule-based sensory organelles which project from the cell surface, enabling detection of mechanical and chemical stimuli from the extracellular environment. It has been shown that cilia are lost in some cancers, while others depend on cilia or ciliary signaling. Several oncogenic molecules, including tyrosine kinases, G-protein coupled receptors, cytosolic kinases, and their downstream effectors localize to cilia. The Hedgehog pathway, one of the most studied ciliary-signaling pathways, is regulated at the cilium via an interplay between Smoothened (an oncogene) and Patched (a tumor suppressor), resulting in the activation of pro-survival programs. Interestingly, cilia loss can result in resistance to Smoothened-targeting drugs and increased cancer cell survival. On the other hand, kinase inhibitor-resistant and chemoresistant cancers have increased cilia and increased Hedgehog pathway activation, and suppressing cilia can overcome this resistance. How cilia regulate cancer is therefore context dependent. Defining the signaling output of cilia-localized oncogenic pathways could identify specific targets for cancer therapy, including the cilium itself. Increasing evidence implicates cilia in supporting several hallmarks of cancer, including migration, invasion, and metabolic rewiring. While cell cycle cues regulate the biogenesis of cilia, the absence of cilia has not been conclusively shown to affect the cell cycle. Thus, a complex interplay between molecular signals, phosphorylation events and spatial regulation renders this fascinating organelle an important new player in cancer through roles that we are only starting to uncover. In this review, we discuss recent advances in our understanding of cilia as signaling platforms in cancer and the influence this plays in tumor development.

## Primary cilia: a snapshot

Cilia are specialized organelles projecting from the surface of most mammalian cells. Primary cilia detect extracellular stimuli, whether chemical or mechanical, and induce a response within the cell [1]. Primary cilia are present in many cell types and are highly diverse, from the long cilia in kidney tubules to modified cilia in retinal photoreceptor cells. Motile cilia function to propel fluids along the surface of the cells such as in the respiratory tract, or to propel the cells themselves as in the case of sperm flagella [1]. Common to all cilia is a microtubule-based axoneme with a nine-fold symmetry arrangement templated on centrioles. Primary cilia typically organize as nine microtubule doublets arranged in a ring (9 + 0), while motile cilia have a central pair of microtubules (9 + 2) that are key for locomotion. In this review, we will focus on primary cilia.

**Fig. 1:**
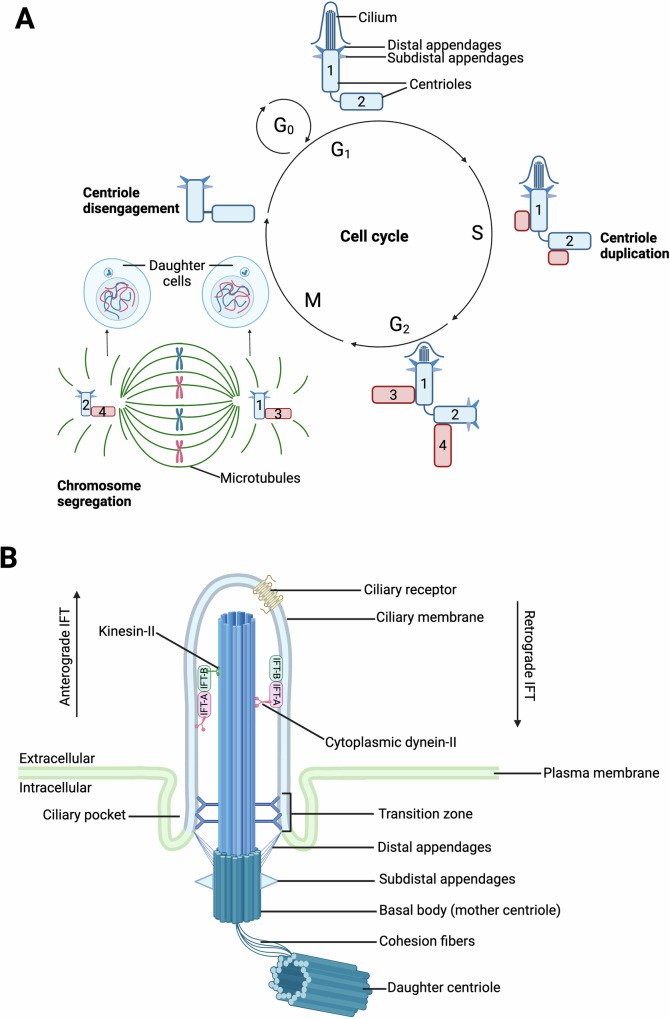
Cilium cycle and structure. **A** In G1 (and G0), the mother centriole [1] and daughter centriole [2] are linked. The mother centriole is modified with distal and subdistal appendages and functions as a basal body, which docks to the plasma membrane. The ciliary axoneme extends from the basal body, protruding from the cell surface. The cilium persists into G2/M-phase but is disassembled prior to cytokinesis. Centrioles duplicate in S-phase, with centrioles [3, 4] growing off the original mother [1] and daughter [2] centrioles. These new centrioles mature during G2-phase. The tether (cohesion fibers) connecting the mother [1] and daughter [2] centrioles breaks at the beginning of M-phase, with the two centriole pairs [1 and 3, 2 and 4] later acting as MTOCs during chromosome separation. Each daughter cell receives a new mother/daughter centriole pair, and the cycle begins again. **B** The primary cilium consists of a microtubule-based axoneme extending from the basal body, a mother centriole modified by distal and subdistal appendages. The cilium is enriched in signaling receptors and effectors. Transport of molecules in and out of the cilium is regulated by the transition zone and mediated by IFT machinery. Created with BioRender.com.

**Fig. 2:**
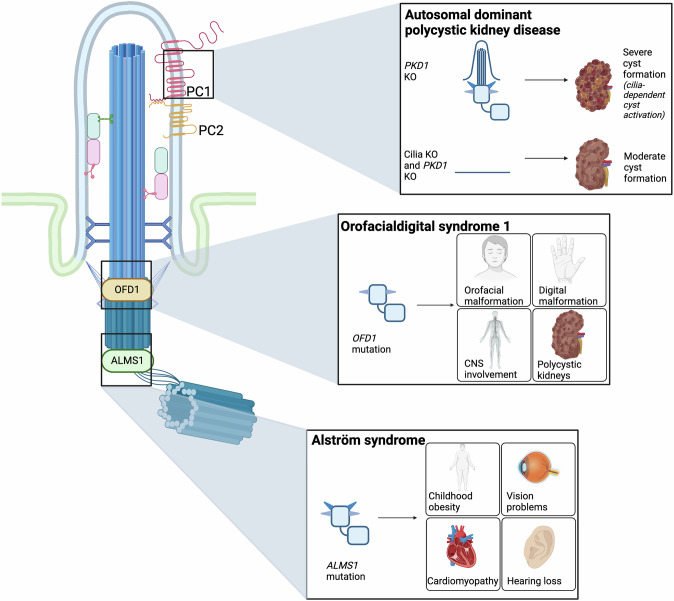
Dysfunction of ciliary proteins leads to a wide range of ciliopathies. Mutations in genes encoding proteins required for ciliary assembly and/or function lead to the development of ciliopathies, such as the above examples of autosomal dominant polycystic kidney disease, orofacialdigital syndrome 1, and Alström syndrome. Despite affecting the same organelle, these mutations affect a variety of organ systems and cause distinct disease phenotypes. Abbreviations: PC1 polycystin 1, PC2 polycystin 2, PKD1 polycystic kidney disease 1, OFD1 orofacialdigital syndrome 1 protein, ALMS1 Alström syndrome 1. *PKD1* is the gene encoding PC1. Created with BioRender.com.

A modified mother centriole, called the basal body, templates the cilium. The basal body features blade-like structures, known as distal appendages (DAPs), which are acquired during G2 and anchor the cilium to the plasma membrane [2]. In eukaryotic cells, in addition to functioning as basal bodies, centriole pairs recruit pericentriolar material to form the centrosome, a microtubule organizing center (MTOC) needed for spindle separation during mitosis. Thus, the primary cilium cycle is closely intertwined with the cell cycle, with cilia typically forming in G1 or G0 and resorbing before or during M-phase [3]. During prophase, the duplicated centrosome separates into two mother/daughter centriole pairs, ensuring only one primary cilium per cell (Fig. 1A). The cilium is enclosed by its own membrane, which is continuous with the plasma membrane but has a distinct protein and lipid composition. A membrane invagination known as the ciliary pocket is also present when cilia are partly intracellular [4]. At the ciliary base, the transition zone functions as a gate, controlling entry and exit of proteins to cilia [5]. Cargo trafficking within cilia is organized by the intraflagellar transport (IFT) system, with kinesin-2 (IFT-B) conveying cargo towards the ciliary tip, and cytoplasmic dynein-2 (IFT-A) organizing traffic towards the ciliary base (Fig. 1B) [6]. Primary cilia are highly enriched in receptors and signaling molecules, and are essential for normal development and cellular homeostasis [1, 7, 8]. Their importance is highlighted by the broad range of genetic disorders that result from mutations in genes that regulate cilia structure and function, diseases collectively known as ciliopathies (Fig. 2) [9, 10].

## The primary cilium as a hub for oncogenic signaling

Primary cilia are hubs for receptors, signaling proteins, and second messengers of a variety of oncogenic pathways [11–15]. This concentration of signaling components in a confined region of the cell places the cilium in an ideal position to facilitate pathway crosstalk, spatiotemporal regulation, and signal amplification. Oncogenic receptors, including Smoothened (SMO), insulin-like growth factor receptor 1 (IGF-1R), epidermal growth factor receptor (EGFR) and platelet-derived growth factor receptor (PDGF-R), can all localize to primary cilia [15–18]. Therefore, it is possible that cilia contribute to cancer by modulating the activity and signaling output of cilia-localized receptors or their downstream effectors. This is supported by two key studies from the Reiter and Alvarez-Buylla labs, showing that subtypes of medulloblastoma and basal cell carcinoma (BCC) with activating SMO mutations are dependent on cilia for tumor formation [19, 20]. Yet, in both cases, they found that tumors driven by constitutively activated GLI2 were accelerated by cilia removal, highlighting the complexity of ciliary involvement in cancer progression [19, 20]. The contribution of other cilia-resident oncogenic molecules to tumor development has been less well studied. For example, AKT, a critical effector of the PI3K pathway and a bona fide therapeutic target in breast cancer, has been shown to localize to the base of cilia [21, 22]. However, whether the presence of cilia in cancer cells may influence the oncogenicity of AKT is unclear. Previously, we have shown that blocking cilia or ciliary signaling can overcome resistance to kinase inhibitors, demonstrating that ciliary regulation of signaling pathways can have a significant impact on cancer cell behavior and response to treatment [18]. Cilia contain extracellular matrix (ECM) receptors such as α2, α3, and β1 integrins, which mediate interactions with the surrounding environment [23]. Integrins can also regulate growth factor signaling pathways [24] and directly interact with growth factors such as FGF1 to form functional signaling complexes [25]. However, whether regulation of these complexes occurs at the ciliary membrane has not been explored.

Regarding mechanical stimuli, fluid flow induces a calcium ion influx in a primary-cilium-dependent manner in kidney epithelial cells [26, 27]. We have shown that the presence of primary cilia plays a role in invasion through the activation of the YAP/TAZ pathway [28]. This pathway also responds to tissue rigidity. Whether cilia can sense and transduce rigidity in the context of an invading cancer cell is an interesting concept that warrants further exploration. In chondrocytes, primary cilia have been shown to elongate and localize to the basal surface when cultured on stiffer substrates [29]. Furthermore, a potential role for cilia in sensing the stiffness of the tumor microenvironment (TME) remains widely unexplored, and could provide insight into the complex involvement of cilia in cancer development and progression. In the context of cancer, the role of primary cilia in detecting chemical stimuli from the external environment has been more intensively studied, and will be discussed further in this review.

### Hedgehog

The Hedgehog (Hh) signaling pathway has been extensively studied in the context of primary cilia. The Hh family contains three proteins in mammals: Sonic Hedgehog (Shh), Indian Hedgehog (Ihh), and Desert Hedgehog (Dhh). Dhh is important for normal sexual differentiation, whilst Ihh and Shh are both critical for normal skeletal development and patterning [30].

**Fig. 3:**
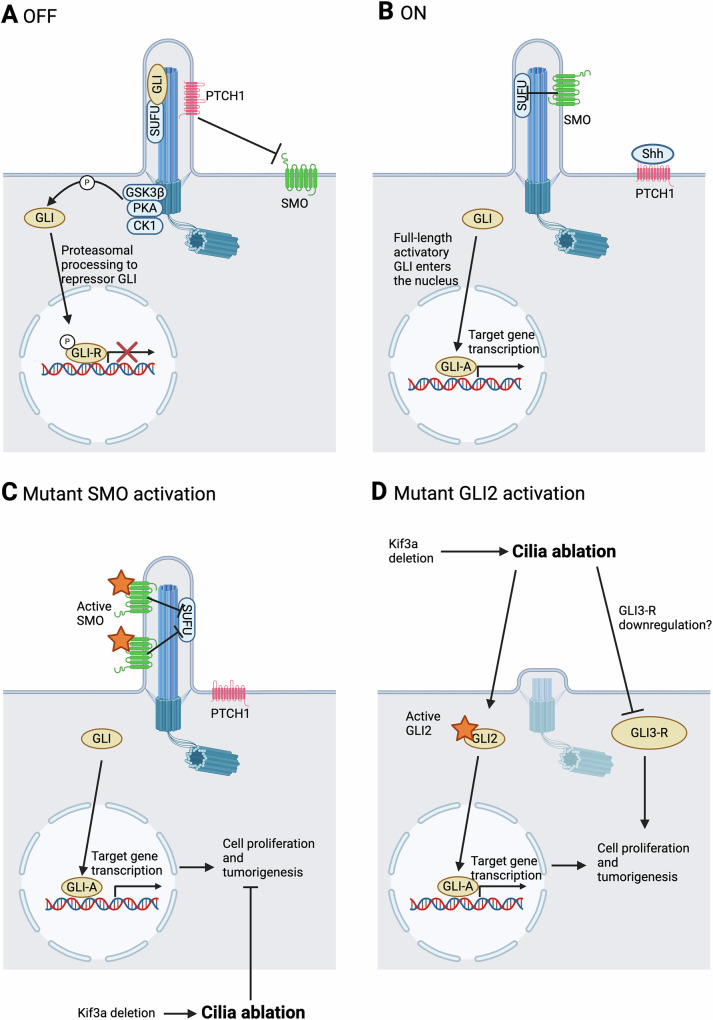
Hedgehog signaling at the cilium in tumorigenesis. **A** In the off state, SMO entry to the cilium is restricted by PTCH1. SUFU sequesters GLI transcription factors at the tip of the cilium. GLI factors are also phosphorylated by a complex of GSK3β, PKA, and CK1, promoting their partial processing to GLI repressors. **B** Upon Hh ligand binding to PTCH1, SMO may enter the ciliary membrane and suppress SUFU, relieving the suppression of GLI. Full-length activatory GLI can enter the nucleus and drive target gene transcription. **C** Models of medulloblastoma and BCC with constitutive ciliary SMO localization show increased cell proliferation and tumor development, which was inhibited by ciliary ablation via KIF3A deletion. **D** Models of medulloblastoma and BCC with constitutively active GLI2 display increased cell proliferation. Tumor development was observed earlier upon cilia ablation via KIF3A deletion, but also occurred in the presence of cilia [19, 20]. Abbreviations: CK1 casein kinase 1, GLI-A activatory GLI, GLI-R repressor GLI, GSK3β = glycogen synthase kinase 3β. Created with BioRender.com.

Hh receptors and downstream effectors localize to cilia in vertebrate cells [16]. In the unstimulated state, the transmembrane receptor Patched1 (PTCH1) localizes to the ciliary membrane and inhibits SMO, a G-protein coupled receptor (GPCR). Suppressor of fused homolog (SUFU) suppresses and sequesters GLI family transcription factors at the tip of the cilium. When phosphorylated by protein kinase A (PKA), GLI2/3 are converted into transcriptional repressors through proteolytic processing (Fig. 3A). Upon Hh ligand binding to PTCH1 at the ciliary membrane, suppression of SMO is relieved, allowing SMO to accumulate in the primary cilium and curb SUFU. GLI2/3 are then released from their repression by SUFU and PKA phosphorylation, promoting their nuclear translocation as GLI- activators, to drive transcription of Hh target genes [31] (Fig. 3B).

Hh target genes regulate the Hh pathway itself (*GLI1, PTCH1, PTCH2*), cell cycle progression (*MYCN, CCND1*), cell survival (*BCL2*), and epithelial-to-mesenchymal transition (EMT; *SNAI1, SIP1*) [32]. These traits are critical in development, but when dysregulated can contribute to a number of the hallmarks of cancer [33] (Fig. 3C, D). PTCH1 mutations are frequent drivers of cancers such as BCC and medulloblastoma [19, 20], and Hh pathway mutations are common across many different cancer types (Table 1). Consequently, a number of Hh-pathway-targeting drugs are currently approved for clinical use in oncology including SMO inhibitors sonidegib and vismodegib (for use in BCC) and glasdegib (for use in acute myeloid leukemia) [34–36]. Additionally, arsenic trioxide, a GLI inhibitor, is approved for use in acute promyelocytic leukemia [37–39].

**Table 1:** The Hh signaling pathway is frequently mutated in human cancers.

Protein	Mutated in	Mutation (nucleotide)	Mutation (amino acid)	Mutation type	Primary or metastatic
SMO	Basal cell carcinoma	1604 G > T	W535L	Missense substitution	Metastatic
	Prostate adenocarcinoma	89 C > T	A30V	Missense substitution	Primary
	Colon cancer	2318 C > G	T773S	Missense substitution	Primary
	Breast invasive carcinoma	481 C > T	R161W	Missense substitution	Primary
	Lung adenocarcinoma	1516 G > A	D506N	Missense substitution	Primary
	Thyroid cancer	409 G > C	D137H	Missense substitution	Metastatic
PTCH1	Basal cell carcinoma	2812 C > T	Q938*	Nonsense substitution	Metastatic
	Prostate carcinoma	3485 T > G	L1162R	Missense substitution	Metastatic
	Colon cancer	3389 C > T	A1130V	Missense substitution	Primary
	Breast invasive lobular carcinoma	1810G > A	E604K	Missense substitution	Metastatic
	Bladder cancer	3944 C > T	P1315L	Missense substitution	Primary
GLI1	Prostate adenocarcinoma	3050 G > A	G1017D	Missense substitution	Metastatic
	Breast invasive ductal carcinoma	214 C > T	R72W	Missense substitution	Metastatic
GLI2	Bladder cancer	3916 G > A	D1306N	Missense substitution	Primary
	Lung adenocarcinoma	1798 C > T	R600C	Missense substitution	Primary
GLI3	Lung adenocarcinoma	1335 C > G	S445R	Missense substitution	Primary
	Pancreatic ductal adenocarcinoma	2623 G > A	R875H	Missense substitution	Primary
SUFU	Breast invasive ductal carcinoma	515 C > T	S172L	Missense substitution	Metastatic
	Thyroid cancer	71delC	P24fs*72	Frameshift deletion	Primary

Sequencing of patient tumor samples reveals that components of the Hh pathway are frequently mutated across different cancer types [44, 172–179].

Ciliary Hh signaling contributes to therapeutic resistance in cancer cells. We have shown that inhibiting GLI or SMO re-sensitizes resistant cancer cells to kinase inhibitors [18]. In a mouse model of small cell lung cancer (SCLC), constitutive SMO activation drove tumor initiation and progression, whilst treatment of SCLC cells with a SMO inhibitor suppressed cell growth [40]. Interestingly, chemoresistant SCLC cells showed increased numbers of primary cilia and increased SMO localization to these cilia, and these cells were more sensitive to SMO inhibition [40]. Collectively, these studies suggest that targeting ciliary signaling alongside conventional therapy could delay or prevent treatment-refractory disease.

### Wnt

Wnt signaling is also critical for normal development, as downstream signaling controls cell proliferation, differentiation, and cytoskeletal organization. The canonical Wnt pathway is switched off in the absence of Wnt ligand when β-catenin is phosphorylated and tagged for proteasomal degradation by the destruction complex, a multiprotein complex mainly consisting of Axin, Adenomatous Polyposis Coli (APC), Casein Kinase 1 (CK1) and Glycogen Synthase Kinase 3 (GSK-3) [41]. When Wnt binds Frizzled (FZD) and low-density lipoprotein receptor-related proteins 5 and 6 (LRP5/6) complexes on the cell surface, Dishevelled (DVL) is recruited to the receptor complex and phosphorylates LRP5/6; this promotes LRP5/6 interaction with Axin, prompting the disassembly of the destruction complex. Subsequently, β-catenin is released from its inhibition and accumulates in the nucleus where it drives target gene expression together with TCF/LEF transcription factors [42, 43]. Dysregulation of the canonical Wnt/β-catenin pathway is common in many cancers, most notably in colorectal cancer [44]. The role of primary cilia in coordinating Wnt signaling is not completely clear. Multiple components of the canonical Wnt pathway localize to the cilium, including β-catenin, LRP6, GSK3β, FZD, and DVL, supporting a possible function for cilia in Wnt/β-catenin signal transduction [45, 46]. Additionally, Foxj1, one of the transcriptional targets of β-catenin is a key regulator of motile ciliogenesis, suggesting that ciliary signaling could also function downstream of this pathway [47]. Kyun et al. have shown that Wnt3a promotes ciliogenesis in RPE-1 and MCF-7/ADR cells, likely due to phosphorylated-β-catenin recruitment to the mother centriole, leading to the reorganization of centriolar satellites [48]. However, a separate study showed that Wnt3a stimulation did not induce ciliogenesis in RPE-1, NIH3T3, or HEK293 cells and that suppressing Wnt ligand secretion did not block cilia formation [49]. Interestingly, the same study showed that DVL knockout did reduce ciliogenesis, perhaps due to the additional role of this protein in the Wnt-planar cell polarity (PCP) pathway, which has been more directly connected with ciliogenesis [49]. Lancaster et al. suggested that β-catenin localization to the basal body restricts canonical Wnt signaling by preventing its nuclear translocation, mediated in part by the ciliopathy protein Jouberin [45]. However, it is not clear how this would account for the total suppression of the pathway. Additionally, cilia depletion experiments in both the mouse embryo [50] and zebrafish [51] showed cilia were not required for canonical Wnt signaling, as cilia ablation did not affect the pathway. Overall, there is a highly complex and incompletely understood relationship between canonical Wnt/β-catenin signaling and primary cilia.

The crosstalk between cilia and non-canonical Wnt pathways seems more clear. The most studied of these non-canonical pathways is the Wnt-PCP pathway, which controls the polarity of cells in the planar axis and cell migration. Wnt ligands bind to FZD and associated co-receptors at the cell surface and trigger a cascade involving the small GTPases Rac1 and RhoA [52]. Downstream cytoskeletal changes then influence cell morphology and polarity [52]. Wnt-PCP signaling is important for the organization and positioning of cilia in a variety of models: primary cilia in radial progenitors and neuroepithelial cells, motile cilia in ependymal cells, and multi-ciliated cells in a *Xenopus* embryo model [53–55]. This relationship between cilia and PCP has been comprehensively reviewed by Wallingford and Mitchell [56]. Cilia and ciliary proteins such as Inversin have been proposed to act as molecular switches controlling activation of canonical and non-canonical Wnt signaling [57, 58]. Mutations in ciliary proteins such as IFT88 and BBS proteins lead to disruption of the PCP pathway in mouse models [59, 60]. This has implications for the role of cilia in cancer, as Wnt-PCP signaling regulates collective cell migration in cancer cells during metastasis [61]. Whether cilia or ciliary signaling have a role in collective cell migration has not been explored.

Recently, Zhang et al. showed that a β-catenin-independent signal could be transduced via ciliary LRP6-coreceptor activation, leading to reduced GSK3 activity and protein phosphatase 1 (PP1) inhibition [46]. Lack of PP1 suppression in cyclin Y and cyclin-Y-like double knockout cells resulted in ciliary defects [46]. While there is no definitive link between Wnt signaling and primary cilia, the Nierhrs lab has recently shown a well-defined link between Wnt signaling and motile cilia biogenesis [62].

While the connection between Wnt signaling and cancer has been well studied, the connection between Wnt signaling and cilia remains ambiguous. Further examination of this connection, which likely involves crosstalk with additional signaling pathways, such as the YAP/TAZ pathway [63], could determine how this relationship may influence cancer progression.

### Hippo

The Hippo signaling pathway plays key roles in controlling cell proliferation, differentiation, and embryogenesis. The MST1/2-SAV1 complex and MAP4K phosphorylate and activate LATS1/2, which phosphorylates the transcriptional coactivators YAP1 and TAZ; this prevents YAP1/TAZ import into the nucleus and interaction with TEAD1-4 transcription factors, thereby restricting gene expression [64]. The regulatory components upstream of YAP1 and TAZ are considered tumor suppressors, whilst YAP1, TAZ, and TEAD1-4 are proto-oncogenes. Target genes of these transcription factors include those involved in proliferation (*CCND1, CCND2, CCND3, MYC*), EMT (*TWIST2, CDH2*), and migration (*CTGF, CYR61*), and hence YAP1/TAZ overactivation has been implicated in the progression of various cancer types [65–67].

NPHP4, a ciliary protein, inhibits LATS1 phosphorylation of TAZ, promoting TAZ nuclear accumulation and gene transcription [68]. In RPE-1 cells, SAV1 was found at the centrosome or basal body in proliferating or ciliated cells, respectively, whilst activated MST1 localized to the basal body in ciliated cells [13]. This study also found that depletion of MST1/2 or SAV1 reduced both the percentage of total ciliated cells and length of cilia, showing that the Hippo pathway can regulate ciliogenesis [13]. Interestingly, ciliary transport proteins IFT88 and IFT20 have been shown to interact with YAP and modulate its activity [69]. Further work would help understand the physiological role of this interaction. We have found that the expression of Spindle assembly abnormal protein 6 homolog (SAS-6) leads to increased ciliogenesis and YAP-mediated cell invasion [28]. Inhibiting ciliogenesis suppressed the SAS-6 invasion phenotype, supporting a potential role for cilia in controlling YAP-mediated invasion [28]. Work by Yang et al. has shown that cilia-mediated Hh signaling promotes smooth muscle development in the embryonic gut, which induces mechanical forces on surrounding mesenchymal cells and drives YAP accumulation in the nucleus, promoting proliferation of the mesenchyme and associated epithelium [70]. The removal of cilia blunted nuclear YAP localization and gut elongation [70].

### Notch

The mammalian Notch signaling pathway is mediated by Notch1-4 receptors and is critical for normal embryonic development. Additionally, it has also been implicated in the development of cancer, promoting EMT, proliferation, and therapeutic resistance [71, 72]. Mechanistically, Delta ligand on the surface of one cell binds to Notch receptors on the surface of another, leading to proteolytic cleavage of the Notch receptor. This cleavage releases the Notch intracellular domain (NICD), which can enter the nucleus and drive target gene transcription via interaction with transcription factors and DNA-binding proteins [73]. Work by Ezratty et al. showed that depletion of cilia via IFT88 and KIF3A knockdown suppressed Notch signaling in an embryonic skin development model [14]. In this setting, presenilin-2, a component of the γ-secretase complex which proteolytically cleaves Notch, localized to basal bodies, and Notch3 localized to the ciliary membrane in cells with activated Notch signaling [14]. In zebrafish, primary cilia have been found to be essential for hematopoietic stem and progenitor cell development, in part via the coordination of Notch signaling [74]. Kong et al. found that Notch signaling primes neural progenitors to respond to Hh signaling, by coordinating the trafficking of Smo and Ptch1 in and out of the cilia, respectively [75]. Notch activation was also reported to increase the length of primary cilia in fibroblasts [75, 76]. The crosstalk between these pathways is enabled by the close association of Hh and Notch pathway components within cilia, allowing for intricate responses to the extracellular environment during development.

### Receptor tyrosine kinases (RTKs)

Active RTKs trigger signaling cascades involved in proliferation and differentiation, notably the MAPK, PI3K, and PLC pathways [77]. As such, overactivation of these pathways, whether through constitutive receptor activation, receptor accumulation, or mutations in downstream effectors, can drive proliferation and tumorigenesis [78]. Amongst others, PDGFRα, EGFR, and IGF-1R are known to localize to cilia and hence may implicate cilia in tumor development [15, 17, 18, 79]. PDGFRα has been shown to localize to cilia, and shows asymmetric accumulation in sister cells after mitosis, localizing to the cilium of the daughter cell inheriting the older mother centriole [80]. How PDGFRα is specifically trafficked to the older cilia, and how cancer cells might benefit from this, is a fascinating question. Similarly, whether constitutively active PDGFRα mutants distinctly localize to cilia, or whether cilia affect PDGFR trafficking and signaling remains to be fully explored. These are clearly relevant and important questions given that dysregulated PDGFRα signaling is associated with poor survival in many cancers [81]. The Christensen, Pedersen, and Satir labs, have extensively followed the links between cilia and PDGFRα signaling, not only in fibroblasts but also during normal embryonic cardiac development [15, 82]. The D842V kinase-active variant of PDGFRα is observed in 5–10% of gastrointestinal stromal tumors (GISTs) [83], and expression of this variant has been shown to promote cilia loss in RPE-1 cells [84]. Interestingly, Hh signaling has been shown to correlate with tumorigenesis in GIST cells [85]. Further work will be required to better understand the complexities of this crosstalk.

Studies in zebrafish and *Xenopus* models found that knockdown of fibroblast growth factor receptor 1 (FGFR1) reduced ciliary length, mediated by a downregulation of *Foxj1, RFX2*, and *polaris* (IFT88) gene transcription [86]. Interestingly, in non-small cell lung cancer, EGFR inhibitor resistance can be mediated by a switch to FGFR dependency [87, 88]. Given the role of FGFR in ciliogenesis [74], this acquired resistance may be partly due to an increase in cilia length and/or changes in ciliary signaling. Supporting this concept, we showed that kinase inhibitor resistant cells had increased ciliogenesis and a switch to FGFR signaling wherein blocking the FGFR pathway suppressed ciliogenesis and re-sensitized cancer cells to kinase inhibitors [18].

### TGF-β receptors

Receptors for TGFβ (TGFβ-RI and TGFβ-RII) also localize to primary cilia. TGFβ-RI/II have been identified at the ciliary tip and base, with an increase in localization at the base following TGFβ stimulation [4]. Additionally, downstream TGFβ effectors SMAD2/3 and ERK1/2 localize to and are phosphorylated at the ciliary base [4]. Stromal TGFβ signaling has been shown to drive colorectal cancer initiation and metastasis [89]. This is an interesting concept, because whilst tumor cells lose primary cilia in some cancer types, stromal cells often retain or increase ciliation [90], suggesting that cilia could still promote tumor progression from the stromal compartment.

### G protein-coupled receptors (GPCRs)

GPCRs are seven-transmembrane domain receptors which are intracellularly attached to heterotrimeric G protein complexes. Upon ligand binding, GPCRs undergo conformational changes which induce exchange of GDP for GTP at the G protein complex, driving the release of the Gα subunit and downstream signaling. A major secondary messenger in GPCR signaling is cAMP, produced by Gα stimulation of adenylyl cyclase at the plasma membrane. Evidence from one of the most common ciliopathies, autosomal dominant polycystic kidney disease (ADPKD), shows that ciliary cAMP drives renal cell proliferation and cyst formation by activation of mTOR, a protein with oncogenic potential [91]. Interestingly, evidence suggests that the cilium contains a distinct pool of cAMP at a higher concentration than the cytosol, but that this is produced in a Gα-independent, PIP3-dependent process [92]. The oncogene SMO, as discussed above, is a known ciliary GPCR involved in the Hh pathway [16]. It has been shown that Shh-mediated SMO activation triggers Ca^2+^ influx into the cilium, which inhibits adenylyl cyclases and subsequent cAMP production, suggesting that ciliary cAMP levels can be regulated in a non-canonical, phospholipid and Shh-mediated manner [92]. Free fatty acid receptor 4 (FFAR4) localizes to cilia in adipocyte progenitors and pancreatic islet cells [93, 94]. FFAR4 has been shown to promote tumorigenic behaviors including enhanced proliferation, migration, and angiogenesis in breast and colorectal cancers, and it would be of interest to know if FFAR4 also localizes to cilia in these cell types [95, 96]. The GPCR melanocortin 4 receptor (MC4R) localizes to cilia in hypothalamic neurons [97], and MC4R mutations are typically associated with obesity, a symptom commonly observed in many ciliopathies, including Bardet-Biedl syndrome and Alström syndrome [9]. However, single nucleotide polymorphisms in the *MC4R* gene have also been associated with increased risk of breast and endometrial cancers independently of their impact on body mass index [98, 99] raising the possibility that this GPCR could be involved in these cancers through novel mechanisms.

## Primary cilia in regulating cancer cell behavior

### Migration and invasion

Signaling molecules localized to and controlled by cilia can influence cellular invasion and motility, two essential processes during embryogenesis and normal development. For example, the endocrine gland derived vascular endothelial growth factor (EG-VEGF) receptor prokineticin receptor 1 (PROKR1) is a GPCR which localizes to primary cilia. PROKR1 has been shown to control trophoblast invasion through activation of ERK1/2 and consequential matrix metalloproteinase (MMP) upregulation, promoting matrix remodeling to allow for embryo implantation during development [100]. Cancer cells hijack EMT programs to acquire more motile and invasive properties critical for metastasis [101]. It turns out that primary cilia can also influence EMT programs, where it has been shown that cilia-mediated Hh signaling induced EMT in cancer cells, and that this differentiation was suppressed in the absence of cilia [102, 103]. Recent work has found that muscle-invasive bladder cancer (MIBC) cell lines may be more dependent on Hh signaling than non-muscle-invasive bladder cancer (NMIBC) counterparts, and that inhibiting Hh signaling reduced EMT in both subtypes [104]. Interestingly, TGFβ treatment was also shown to promote EMT, migration, and invasion of bladder cancer cells, with a concomitant upregulation of Hh, suggesting crosstalk between these two pathways [105]; whilst primary cilia were not investigated in these studies, components of both Hh and TGFβ pathways localize to cilia [4, 16]. In bladder cancer, immunohistochemical analysis of patient biopsies has revealed the presence of cilia in healthy bladder, MIBC, and NMIBC cells [106]. In NMIBC samples, primary cilia were identified on cells crossing the basement membrane and in proximity to blood vessels, suggesting a role for ciliated cells in local stromal invasion [106]. Hh pathway components Gli1 and SMO were increasingly present in both tumor types, but more prominently in MIBC, suggesting a correlation between cilia-dependent Hh signaling and tumor progression [106].

It has been reported by multiple groups that breast epithelial cells lose primary cilia throughout cancer progression, with the suggestion that the loss of cilia is an early event in breast tumorigenesis [107, 108]. However, these studies rely on the use of acetylated tubulin as a ciliary marker and would be strengthened by additional ciliary-specific markers such as the ciliary membrane marker ARL13B [109, 110]. Even in these studies it was noted that cilia were present in some cases: Yuan et al. observed low numbers of cilia in both cell lines and patient tissue samples of the basal B subtype, and Menzl et al. found that ciliated cells from invasive breast cancer samples often expressed CK5, a marker of the basal subtype and also of poor prognosis [107, 108]. This suggests that the presence of primary cilia in breast cancer may be subtype-specific, and that they may play a particular role in the aggressive basal-like molecular subtype. We have recently shown that overexpression of the centriolar protein SAS-6 increases ciliogenesis in RPE-1, HMEC, and MCF10AT1 cells, the latter being a model for early breast tumorigenesis [28]. Intriguingly, *SASS6* overexpression also induced increased invasion, which was found to be cilia- and YAP-dependent. SCL/TAL1 interrupting locus (STIL) is another centriolar protein essential for centriole and cilium formation and a close interactor of SAS-6 [111]. STIL overexpression has been shown to promote migration and invasion in triple negative breast cancer cells [112].

Some contrasting evidence shows that cilia loss in kidney tubule cells drives EMT, and that this effect is enhanced by the addition of TGFβ, a cytokine commonly found in the fibrotic TME [113]. In epicardium-derived cells, evidence shows that loss of cilia can either promote or suppress EMT [114, 115]. This unclear role for cilia in EMT may be explained by the fact that EMT is a spectrum, rather than a binary process [116, 117], and that cilia may be involved in the formation of an intermediate-like state or partial-EMT.

### Cancer stem cells

Cancer stem cells (CSCs) are implicated in tumor initiation and recurrence, as they have an innate ability to resist treatment and self-renew, leading to the restoration of a resistant tumor mass following therapy [118]. CSCs are dependent on EMT programs to develop stem-like properties, displaying high degrees of cell plasticity. Primary cilia are known to be essential for normal stem cell differentiation and embryonic development, as illustrated by the number of ciliopathies which occur upon dysfunctional ciliary gene expression, including organ laterality defects, skeletal abnormalities, and congenital heart defects [9]. This raises the question of whether primary cilia are involved in regulating signaling pathways used by CSCs to drive tumor initiation and treatment resistance. In mesenchymal stem cells, the removal of cilia has been shown to downregulate the expression of stemness-related genes *OCT4*, *SOX2*, and *NANOG* [119]. In muscle stem cells, cilia have been shown to be critical for self-renewal and expansion [120]. However, cilia have also been shown to be dispensable for self-renewal in human pluripotent stem cells, but to later contribute to differentiation of neural rosettes [121]. This evidence suggests that cilia can play roles in the self-renewal and/or terminal differentiation of stem cells, perhaps in a tissue-specific manner.

Recent work by Guen et al. has shown that primary cilia control the stemness of mammary stem cells (MaSCs) and tumor-initiating cells (MaTICs) following the induction of EMT programs [103]. This study established a hierarchy, where EMT induced primary ciliogenesis, and the subsequent increase in Hh signaling supported the stem-like properties of basal MaSCs and MaTICs. Critically, they showed that removing cilia in MaTICs greatly reduced or even abolished their tumor-initiating capacity in an orthotopic implantation model [103]. Following this, Wilson et al. demonstrated that the transcription factors Snail and Zeb1 not only induce expression of EMT markers such as N-cadherin and vimentin, but also ciliogenesis inducers such as FGFR1 [102]. They also found evidence that GLIS2, a transcriptional repressor of the Gli–similar (GLI-S) family of Krüppel-like zinc finger transcription factors [122], may be inactivated by BBS11 at the base of primary cilia in mammary epithelial cells, hence increasing stem-like characteristics [102]. Lee et al. have shown that Kelch domain containing 8 A (KLHDC8A) is specifically upregulated in glioblastoma stem cells (GSCs), compared with differentiated controls, and promotes their growth [123]. Interestingly, KLHDC8A also promotes ciliogenesis and increased Hh signaling specifically in GSCs while ablation of cilia reduced GSC proliferation, further implicating cilia in cancer stemness [123].

### Therapeutic resistance

Cilia have also been implicated in the cancer cell response to therapy beyond their contribution to stemness. Our group previously investigated the role of cilia in various cancer cell line models resistant to kinase inhibitors or chemotherapeutic agents. We found that both acquired and de novo resistant cells showed more cilia, longer cilia, and increased Hh signaling [18]. Critically, this work showed that removing cilia or inhibiting components of the Hh pathway sensitized cells to kinase inhibitors, showing the potential therapeutic implications of targeting primary cilia or their associated signaling pathways [18]. In agreement with these findings, it has been shown that pancreatic cancer cells, which typically lose primary cilia during tumor progression, regrow cilia following treatment with cisplatin, gemcitabine, and etoposide [124]. Disruption of cilia was shown to increase sensitivity to cisplatin in pancreatic cancer both in vitro and in an in vivo xenograft model [124]. The increase in cilia in cisplatin-treated PANC-1 cells was in part attributed to the ATM- and ATR-mediated DNA damage responses activated by cisplatin treatment. ATM and ATR activation led to excessive formation of centriolar satellites and activated autophagy, which induces ciliogenesis and therefore chemoresistance of these cells [125, 126]. In glioblastoma cells, treatment with temozolomide or ionizing radiation induced ciliogenesis, and disrupting ciliogenesis sensitized cells to these treatments [127, 128]. In agreement with the above findings in pancreatic cancer, autophagy and the DNA damage response were activated in glioblastoma cells following these treatments. Interestingly, IR-induced activation of DNA-PK and ATM was diminished by IFT88 knockdown, suggesting a potentially bi-directional interplay between cilia and the DNA damage response [127].

**Fig. 4:**
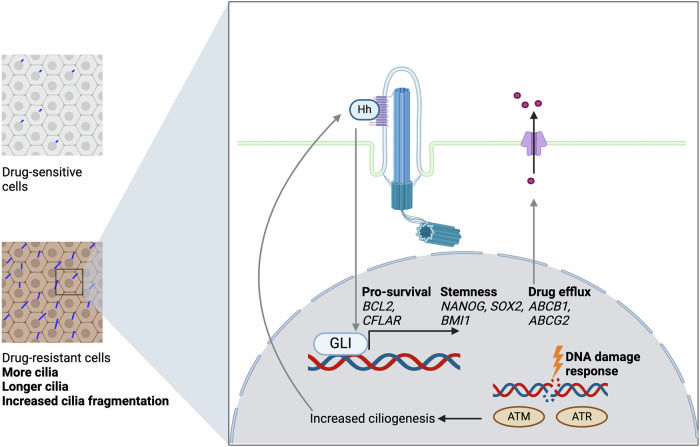
Cilia and ciliary signaling contribute to therapeutic resistance. Ciliary Hh signaling promotes the transcription of genes associated with therapeutic resistance, including those promoting survival, stem cell behavior, and drug efflux. Ciliogenesis-promoting pathways, such as activation of the DNA damage response upon chemotherapeutic treatment, enhance ciliary signaling and consequently drive resistance. Created with BioRender.com.

Overall, the evidence suggests that primary cilia contribute to therapeutic resistance in several ways (Fig. 4). They coordinate pro-survival signaling pathways such as the Hh pathway, identifying the possibility of combining Hh inhibitors with conventional treatments to reduce resistance [32]. On the other hand, it has been shown that cancer cells can also become resistant to Hh pathway inhibitors by losing cilia to render them independent of SMO signaling [129]. As described above, cilia are also linked to an activated DNA damage response, another mechanism of treatment resistance [124, 127]. It has been argued that the presence of cilia may denote a more quiescent cell population that is more resistant to chemotherapies that target fast-cycling cells [130, 131]. However, this is conflicted by the fact that embryonic stem cells have cilia and rely on ciliary signaling throughout development, during which these cells are actively proliferating [1, 120, 132]. Cilia have also been shown to persist after G1/S and into M-phase, and so, are not necessarily indicative of a quiescent population [3]. In our experimental system, increased resistance could not be attributed to any changes in cell cycle profile [18]. Finally, cilia may also drive resistance by promoting a CSC phenotype, as CSCs are known to display innate therapeutic resistance, for reasons such as an increase in drug efflux pump activity, enhanced evasion of apoptosis, and the adoption of a more mesenchymal phenotype [133, 134].

### Interaction with the tumor microenvironment

Primary cilia mediate interactions between cancer cells and the surrounding TME, enabling adaptation of the TME to advance tumor progression. This is particularly important in metastasis, where distant locations must be primed for tumor cell colonization. It has been shown that gastric cancer cells induce ciliogenesis in mesenchymal stem cells, associated with abnormal Wnt signaling, and inhibit osteoblast proliferation [135]. This dysregulated ciliary signaling pathway results in bone loss, potentially priming for metastatic colonization. Bone metastasis also occurs in both breast and prostate cancer, where primary cilia have also recently been implicated in the crosstalk between osteocytes and tumor cells [136]. In early metastasis, osteocytes secrete TNFα, mediated by IFT88 of their primary cilia, which suppresses tumor cell proliferation. The authors of this study suggest that this encourages migration until the tumor reaches a critical mass, at which point tumor-secreted TGFβ disrupts osteocyte cilia and associated TNFα secretion. Tumor cells can then rapidly proliferate following the relief of the TNFα-mediated suppression [136].

Cancer-associated fibroblasts (CAFs) are prominently found in the TME of most epithelial tumors. CAFs enhance tumor progression by secreting growth factors and a fibrotic ECM, activating oncogenic signaling pathways and providing a physical barrier for immune cell infiltration [137]. In an in vitro breast cancer model, it was shown that co-incubation of breast cancer cells with adipose progenitor cells drives adipose progenitors to produce TGFβ and differentiate into CAFs, in a cilia-dependent manner, suggesting that cilia can shape the TME [138]. Conversely, colonic fibroblasts have been shown to lose primary cilia throughout colon carcinogenesis, attributed in part to IL-6 signaling [139]. Mice with colonic fibroblasts lacking cilia were more sensitive to colonic inflammation and developing colitis and colitis-associated carcinogenesis [139]. These examples show the complexity of the role of cilia in shaping the TME, as either dependence on or loss of cilia can drive tumor progression depending on disease context. Cilia-mediated interactions may drive a constant crosstalk between tumor and stromal cells, shaping the behavior of both cell types, leading to dynamic effects which may support or suppress tumor progression. In pancreatic ductal adenocarcinoma (PDAC), a tumor with a devastating prognosis, cilia are lost from tumor cells as the disease progresses [140, 141], whilst ciliogenesis increases in the associated stroma [90]. In pancreatic β-cell models, cilia have been shown to make physical and functional contacts with other cells [142]. At the onset of chemotherapy resistance, PDAC cells regain cilia [124]. Whether cancer cells promote ciliogenesis in the stroma or whether signaling from stroma promotes ciliogenesis in resistant cells are important questions. How could these signals be transduced? Ciliary tips can be actively shed and contain signaling molecules and transmembrane receptors [143]. We have found that drug-resistant cancer cells shed ciliary tips [18]. Work by the Sarkisian lab concluded that ciliary vesicles may support intercellular communication and contribute to pathogenesis in glioblastoma multiforme [144]. Whether and how ciliary vesicles might aid intercellular communication amongst cancer cells and/or the tumor stroma to elicit a biological response are fascinating questions in both the cancer and cilia fields.

It is likely that the stiffness of the cancer microenvironment also triggers signal transduction via primary cilia, which in the context of cancer may be found on tumor or stromal cells. Signaling at the cilium can induce changes in the microenvironment, for example by impacting the expression of MMPs, enzymes which degrade and remodel the surrounding ECM. As mentioned above, PROKR1 activation by EG-VEGF at trophoblast cilia induces MMP2 and MMP9 expression via ERK1/2 activation [100]. In chondrocytes under tensile strain, ERK1/2 activation leads to the downregulation of MMP1 and MMP13, a process which was found to be cilium-dependent [145]. Additionally, IFT88 knockdown in human dermal fibroblasts increased MMP1 expression [146]. This evidence suggests that cilia could play key roles in the remodeling of the non-cellular components of the TME.

Immune cells also make up a major proportion of the TME, playing both pro- and anti-tumorigenic roles. Whilst immune cells are believed not to form cilia, there are remarkable similarities between the immunological synapses formed between cytotoxic T lymphocytes and their target cells [147, 148]. The cytoskeletal reorganization and alteration of membrane content at the synapse mimics the formation of primary cilia, with the most striking similarity being the mother centriole docking to the membrane via centriole distal appendages [149, 150]. It has been suggested that these cells form a ‘frustrated cilium’, where centrioles anchor to the membrane, coordinating the localization of receptors and effectors in close proximity, but no axoneme extends to form a cilium [150–152]. In support of this, it has been shown that SMO and PTCH1 traffic towards the immunological synapse upon recognition of a target cell, and intracellular Ihh signaling is required for centrosome polarization and cytotoxic granule release [148]. Treatment with small molecule inhibitors or genetic ablation of SMO suppressed actin clearance and centrosome polarization at the immune synapse, thereby decreasing cytotoxic T cell-mediated killing [148]. This is an important consideration for cancer therapies, as Hh pathway inhibitors will impact both tumor cells and the surrounding TME, in this case limiting the immune response.

### Cellular metabolism

The involvement of cilia in regulating cellular metabolism can be inferred from the obesity phenotype observed in many ciliopathies, such as Alström syndrome and Bardet-Biedl syndrome [9]. However, more direct molecular evidence now shows that cilia can regulate metabolism of many substrates. This has interesting implications for cancer progression, as dysregulated cellular energetics is recognized to be one of the hallmarks of cancer [33].

Perhaps the most famous example of dysregulated metabolism in cancer is the Warburg effect, where cancer cells switch towards aerobic glycolysis in favor of mitochondrial oxidative phosphorylation [33]. In thyroid cancer cells, loss-of-function mutations in IFT88 or KIF3A promoted glycolysis and dysfunctional mitochondrial metabolism, confirming that primary cilia could influence the Warburg effect in cancer cells [153]. This could perhaps be explained by ciliary Hh signaling – SMO activation has been shown to switch adipocyte metabolism towards glycolysis via AMPK activation [154]. This glycolytic shift was found to be cilium-dependent, as cilia-deficient mouse embryonic fibroblasts (MEFs) showed severely blunted glucose uptake and lactate secretion upon SMO activation compared to control cells [154]. A more rigid ECM has recently been shown to induce a shift towards glycolytic metabolism in PDAC, mediated by Chloride intracellular channel 1 (CLIC1) [155]. Nuclear translocation of the WNT effector TCF4 leads to increased CLIC1 expression, and subsequent stabilization of HIF1α, driving aerobic glycolysis [155]. Interestingly, CLIC5b, another member of the family, localizes to cilia and regulates ciliogenesis in zebrafish [156]. Whether CLIC family proteins promote a functional crosstalk between ECM sensing, ciliary signaling, and metabolism has never been been considered.

The amino acid glutamine supplies carbon and nitrogen to fuel the TCA cycle and the biosynthesis of lipids, amino acids, and nucleotides. Consequently, many cancer cells display an ‘addiction’ to glutamine [157]. Steidl et al. have shown that cilia-deficient MEFs show significantly reduced glucose and glutamine consumption compared to control cells [158]. In this study, removal of glutamine from the media also drove cilia elongation, showing that cilia can respond to glutamine and alter its metabolism [158]. This is proposed in part to be due to asparagine synthetase, the enzyme responsible for converting aspartate and glutamine to asparagine and glutamate, which localizes to and acts at the base of the cilium [158].

### Cell cycle regulation

Primary cilia are tightly regulated by the cell cycle, due to the dual role of centrioles in both basal body and centrosome formation. Given that cilia could represent a physical barrier for mitosis, it has been assumed that the presence of cilia could negatively affect cell cycle progression. Accordingly, it has been shown that depletion of the centrosomal protein Nde1 in NIH3T3 cells and zebrafish embryos caused elongation of cilia and cell cycle re-entry delay [159]. This cell cycle delay was found to be cilium-dependent, as knockdown of IFT88 or IFT20 in NIH3T3 cells inhibited cilia formation and reversed the delayed entry into S-phase observed upon Nde1 depletion [159]. Another study showed that Tctex-1, a component of the cytoplasmic dynein light chain, coordinates S-phase re-entry in ciliated cell lines (RPE-1, NIH3T3) but not in non-ciliated cells (HeLa, COS-7), suggesting that this control of the cell cycle is cilium-dependent [160]. Phospho(T94)Tctex-1 was found to localize to the transition zone of cilia prior to ciliary resorption, whilst Tctex-1 knockdown delayed ciliary disassembly [160]. Aurora kinase (AURKA), a mitotic kinase which activates CDK1-cyclin B, interacts with HDAC6 at the basal body, promoting deacetylation and subsequent disassembly of the cilium [161]. Further research has shown that trichoplein localizes to centrioles in proliferating cells and activates AURKA [162]. AURKA or trichoplein knockdown both induced cell cycle arrest, but this was reversed by knockdown of IFT20, suggesting that ablation of cilia can drive cell cycle progression [162]. A common belief about the role of cilia in cancer is that for tumors to progress, cells must lose their cilia to relieve a ‘brake’ on proliferation. Cilia loss does appear to occur in several cancer types throughout tumorigenesis, including breast, prostate, and bladder cancers [107, 163, 164]. However, the idea that loss of cilia promotes proliferation is perhaps oversimplified, and is gradually being reconsidered due to the increased use of additional ciliary-specific markers such as ARL13B, recent advances in electron microscopy techniques [85, 106] and better knowledge surrounding cilia and cancer.

First, many cancer cells are proliferative but do still retain their cilia, as seen in medulloblastoma, BCC, bladder cancer, lung cancer and glioblastoma, among other examples [19, 20, 106, 165]. In PDAC, loss of primary cilia does not result in changes in cell proliferation [140]. There is conflicting evidence for whether certain tumors retain cilia, such as in glioblastoma, where different groups have reported that tumors lose cilia, display defective cilia, or consistently display cilia [165–167]. Whilst these differences may represent intra-patient heterogeneity, they may also be caused by differences in culture and fixation conditions. As cilia are very sensitive to growth factors and serum, any minor differences in media composition, for example, may account for these differences. This highlights the importance of using models which are as physiologically relevant and robust as possible, especially when studying a dynamic organelle such as the primary cilium. In addition, while proteins such as IFT88 or IFT20 have a defined function in ciliogenesis, they have been shown to have cilia-independent functions [168]. This is particularly relevant for IFT88, a key component of the IFT system. IFT88 has also been shown to inhibit Che1, an inhibitor of the Rb tumor suppressor – upon IFT88 ablation, Che1 then inhibits Rb, freeing E2F transcription factors to drive cell cycle progression [169]. IFT88 depletion has also been shown to induce spindle misorientation and mitotic defects in cell and zebrafish models, attributed to disrupted microtubule trafficking [168]. For these reasons, any increase in cell cycle progression upon cilia ablation must be closely examined to rule out any potential non-ciliary functions of the perturbed cilia-promoting protein (e.g., IFT88). Whether blocking later stages of ciliogenesis might affect the cell cycle or whether this is due to extra-ciliary functions of cilia proteins remains a critical question in the field.

ADPKD is one of the most common ciliopathies, affecting ~1/1000 births. The majority of ADPKD cases are caused by mutations in *PKD1*, encoding polycystin 1 (PC1), with a minority of cases being caused by *PKD2* mutations encoding polycystin 2 (PC2) or other mutations [170]. ADPKD provides further evidence for the complex relationship between cilia and cell cycle control. ADPKD is characterized by fluid-filled cyst formation on renal tubules, leading to complications such as hypertension, recurrent infections, and ultimately kidney failure. Intriguingly, cyst formation occurs upon PC1 loss or cilia loss, yet cystogenesis is most severe when PC1 is lost but cilia remain, in a process known as cilia-dependent cyst activation (CDCA) [171]. As cyst formation is at least partly attributed to increased proliferation of renal tubule cells, this provides some disease-relevant evidence that loss of cilia is not required for enhanced proliferation.

## Conclusion

Cilia are remarkable organelles that we are only now beginning to understand. In this review, we have examined how cilia and ciliary signaling can influence various cancer phenotypes. Cilia are rich in receptors and effector molecules that pack together tightly in a small space, which increases the signal/surface ratio. This concentrated signaling could have dramatic consequences on oncogenic effectors such as Akt, which functions downstream of multiple ciliary signaling pathways and localizes to the ciliary base, and therefore could foster pathway crosstalk. We hypothesize that cilia might modulate cancer by controlling the activation of ciliary receptors and downstream effectors. This control could involve functional specificity, spatiotemporal regulation, and/or signal amplification.

**Fig. 5:**
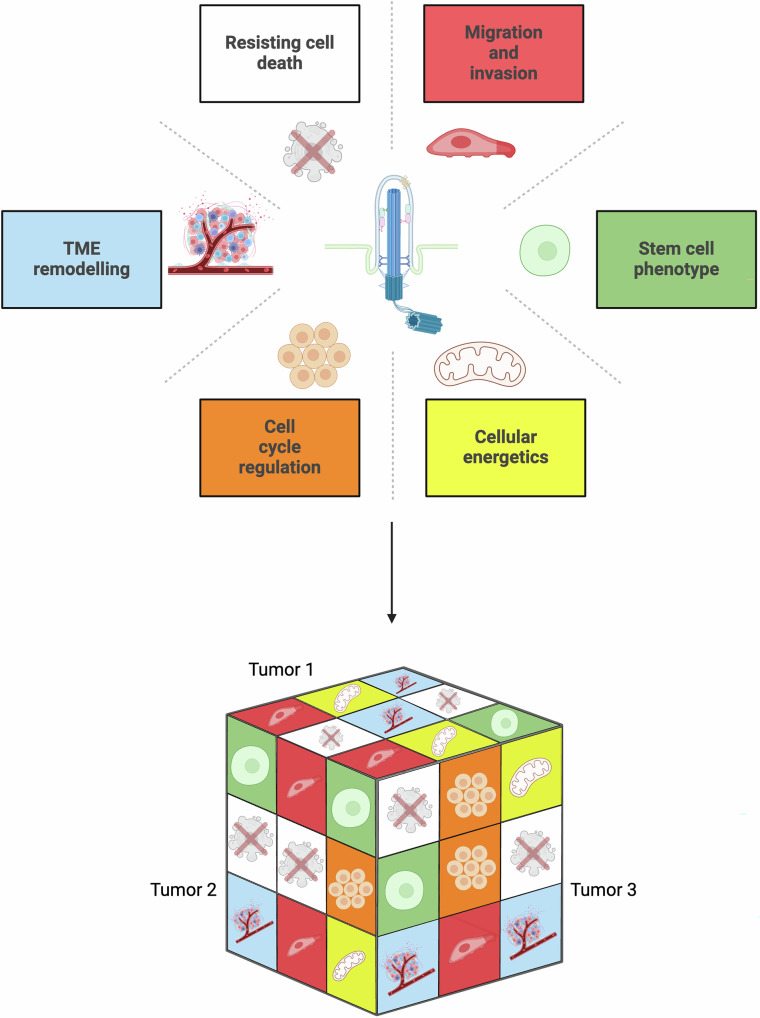
The many faces of cilia in cancer. Primary cilia have been implicated in many of the hallmarks of cancer [33]. Tumors can display different dependencies on cilia and ciliary signaling, requiring investigation into individual patients and subtypes. Cilia could represent a novel target to restrict cancer progression in those tumors which are dependent on ciliary signaling. Created with BioRender.com.

While not long ago, cilia were thought to be dispensable for cancer, our lab and other groups have demonstrated a key role for cilia in cancer development and treatment resistance. Lack of cilia has also been shown to promote resistance by disallowing Hh pathway specific inhibition. Thus, evidence indicates that the role of cilia in cancer is complex, and needs to be contextualized. What is clear is that cilia can control key elements of the ‘hallmarks of cancer’. In this regard, how cilia can modulate oncogenic potential likely depends on the particular cellular and molecular features of a cell at the onset of transformation and/or progression, which define the many faces of cilia in cancer (Fig. 5).

## Glossary

*Axoneme* – core of the cilium, a ring of nine microtubule doublets

*Basal body* – a modified mother centriole at the base of the cilium from which the axoneme extends

*Centriole* - barrel-shaped organelle composed of nine microtubule triplets

*Centrosome* – a pair of centrioles arranged perpendicularly and surrounded by pericentriolar material

*Ciliary membrane* – membrane surrounding the axoneme with a unique protein and lipid composition different to that of the plasma membrane

*Ciliary pocket* – membrane invagination found surrounding the base of partly intracellular cilia

*Daughter centriole* – centriole assembled at least 1.5 cell cycles after the mother centriole it is paired with, not modified with distal or subdistal appendages

*Distal appendages* – structures protruding from the distal end of the mother centriole which mediate docking to the plasma membrane during ciliogenesis

*Intraflagellar transport* – transport of cargo along the axoneme, either towards the ciliary tip (anterograde, mediated by dynein-2) or towards the cell body (retrograde, mediated by kinesin-2)

*Microtubule organizing center* – site of microtubule nucleation and organization

*Mother centriole* – centriole assembled at least 1.5 cell cycles prior to the daughter centriole it is paired with, modified with distal and subdistal appendages and has the capacity to become a basal body

*Pericentriolar material* – a dense matrix of proteins surrounding the centrioles

*Subdistal appendages* – structures protruding from mother centriole, further from the distal end of the centriole than distal appendages

*Transition zone* – a zone at the base of the cilium which controls entry/exit of proteins into the cilium
